# Calcium phosphate ceramics as bone graft substitutes in filling bone tumor defects

**DOI:** 10.4103/0019-5413.39588

**Published:** 2008

**Authors:** KC Saikia, TD Bhattacharya, SK Bhuyan, DJ Talukdar, SP Saikia, P Jitesh

**Affiliations:** Department of Orthopedics, Guwahati Medical College and Hospital, Guwahati, Assam, India

**Keywords:** Benign bone tumor, bone graft substitute, hydroxyapatite, calcium phosphate ceramic

## Abstract

**Background:**

Synthetic bio-inert materials are currently used as an alternative to autogenous bone graft. Calcium hydroxyapatite (HA) and Beta tri-calcium phosphate (β-TCP), which belong to the calcium phosphate ceramics group, are biocompatible and osteo-conductive. The purpose of this study is to analyse the use of HA and β-TCP in their ceramic forms as a bone graft substitute in filling bone voids after curettage of benign bone tumors.

**Materials and Methods:**

Twenty-four patients in the age range of 3.5-55 years (mean 14.3 years) having benign bone tumors with bone defects were filled with bone graft substitute following curettage. In 20 patients bone defects were filled with block/granules of HA ceramic and in four with β-TCP. Fibular strut graft was packed with HA in four patients. The patients were followed up for an average of 18 months (range 12-36 months).

**Results:**

The functional status of the patients at follow-up was evaluated and compared with preoperative functional status. Early incorporation of graft substitutes became evident radiologically between 6 and 10 weeks (Stage I). Complete incorporation (Stage III) was observed in an average of nine months (6-18 months). Clinical healing was observed before radiological healing. The average time taken to return to preoperative function was 14 weeks. There was no recurrence of lesion or growth retardation.

**Conclusion:**

Calcium hydroxyapatite and β-TCP are excellent bone graft substitutes for autogenous bone graft in filling voids after curettage of benign bone tumors.

## INTRODUCTION

Bone grafts are often necessary to provide support, fill voids and to enhance biological repair of skeletal defects. Autogenous cancellous bone graft, usually from the iliac crest has been considered the gold standard because of its osteo-conductivity, osteo-inductivity, osteogenic potential and lack of immunogenicity as well. This standard auto graft has its own share of problems like inadequate amount, especially in children, donor site morbidity and potential complications like pain, hematoma, infection, etc.^1^,^2^ To overcome these problems, biological alternatives, mainly allografts and xenografts have been processed and used. But limitation of ready availability, high cost and problems of immunogenicity have accelerated the search for synthetic, bio-inert materials as an alternative. Calcium hydroxyapatite (HA) and beta tri-calcium phosphate (β-TCP), which belong to the calcium phosphate ceramics group, are bio-compatible osteo-conductive materials which offer a chemical environment and a surface conducive to new bone formation.^3^–^6^ Their efficacy as substitutes for autologous graft in filling bone defects have been proved by various clinical as well as experimental studies. Hydroxyapatatite (HA) can be coral based or chemically derived synthetic ceramic Ca_10_ (PO_4_)_6_ (OH)_2_. Coral-based ones are more dense and are commonly used for dental purposes. The exoskeleton of marine species Goniospora yields coral of 500 μ size. This is more commonly used in orthopedic practice. Synthetic HA is formed by the precipitation of calcium nitrate and ammonium dihydrogen phosphate. From the clinician's point of view, it is important to note that the Ca-P ratio, particle and pore sizes vary from product to product. No clinical or experimental study is available for G-bone. The HA is radio-opaque and can be re-sterilized. The TCPs are more quickly biodegradable than HAs. The HA has good mechanical properties. The acceptance of these substitutes by host tissues is determined by two important features - pore diameter and the porosity or inter-connectivity. Minimum pore size of 100 μ is optimal for bone in-growth^7^, whereas pore sizes more than 200 μ facilitate development of mature osteon. Inter-connectivity is essential because dead-end pockets limit vascular supply to the in-growing bone. The form, whether blocks or granules, can also determine the clinical utility as the size of the bone defect determines the appropriate size of the implant. In metaphyseal large voids, a block form of porous HA or TCP actually provides better support.

This is a prospective study to evaluate the role of HA and β-TCP in their ceramic form as a bone graft substitute in filling voids after curettage of benign bone tumors.

## MATERIALS AND METHODS

From January 2003 to May 2006, either HA or β-TCP were used as bone graft substitute to fill the voids after intra-lesional curettage in 24 confirmed cases of benign bone tumors. The inclusion criterion was:benign bone tumors confined to the normal anatomical limits of the bone with or without pathological fracture and the exclusion criteria were (i) Very large benign tumors, (ii)Active contiguous infection, (iii)Suspected or diagnosed malignant lesions, (iv) Traumatic bone loss. Fourteen of them were female and 10 were male. The age ranged from 3.5-55 years (avg. 14.3 years). All patients underwent clinico-radiological evaluation and the diagnoses were confirmed by aspiration cytology or open biopsy. The lesions were fibrous dysplasia (*n* = 8), simple bone cyst (*n* = 7), aneurysmal bone cyst (*n* = 4), eosinophilic granuloma (*n* = 2), giant cell tumor (*n* = 1) and chondromyxoid fibroma (*n* = 2).

The HA used in our study were (i) HA of bovine origin, marketed in India as “G-bone” (Surgiwear) blocks and (ii) the synthetic one, “Orthogran” (Top-Notch) granules. Granule size was 2-3 mm with pore size of 200-300 μm. Large voids in the metaphyses were filled with HA blocks/granules as degradation is slow in HA. Small defects in the diaphyses were filled with β-TCP, as bio-degradation is earlier in β-TCP. The shape appropriate for clinical application was chosen or a combination of these was used to achieve compact packing of the defect. The granule form of synthetic β-TCP with pore size of 100-500 *μ*m was used (Synthes- “ChronOS”).

**Figure 1:**
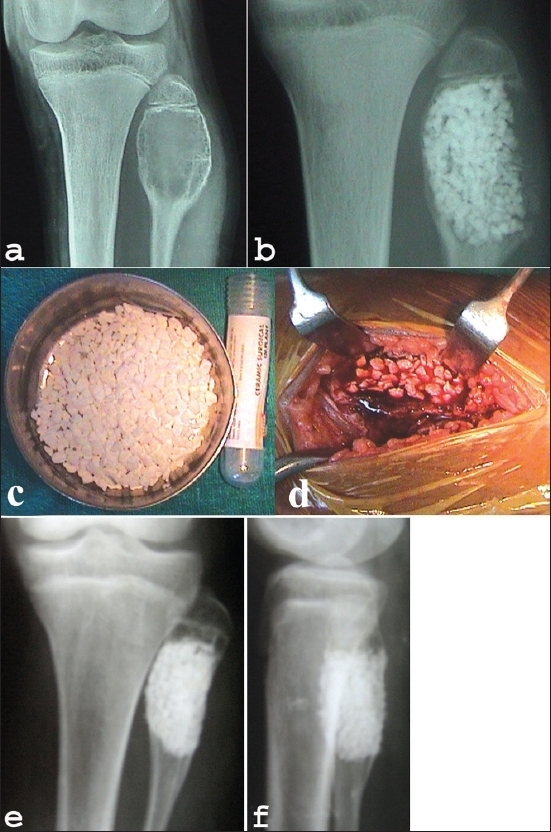
(a) Preoperative x-ray of knee AP view (a) shows a cavitary lesion (aneurysmal bone cyst) of upper end of fibula in 14 year old girl. Immediate postoperative x-ray (b) shows the cavity filled with HA granules. (c) and granule filled cavity (d) Peroperative clinical photograph of same patient showing HA granules (d). (e) Anteroposterior and lateral x-rays (f) of same patient at 2 yrs follow-up shows remodelling.

Standard surgical principles and approaches were used to expose the site. The cortical windows in the defects were made just large enough to facilitate thorough intra-lesional curettage with minimal disturbance to the periosteum [Figure 1a–d]. Approximate volume of the lesion was assessed by insinuating sterile roller gauze into the cavity.^4^ Mean volume of the defects was 26.2 cc (10-70 cc). No adjuvant was used after curettage. The grafts were mixed with autologous blood from the operative field prior to application. After packing the defect, either the periosteum was meticulously re-apposed or in situations where periosteal flap coverage was inadequate, careful soft tissue coverage was done to prevent extravasations of the graft. Check X-rays were done to confirm compact packing (complete packing of the cavity without keeping any unpacked areas in the voids). In eight cases autogenous cancellous bone graft harvested from the iliac crest and/or contralateral fibular graft was used along with the calcium phosphate material. In one case of pathological fracture of the proximal femur, 95° condylar blade plate fixation was done. Postoperative immobilization was individualized according to the site, size of the lesion and the usage of internal fixation. The patients were followed both clinically as well as radiologically at three, six, 12 and 18 weeks and subsequently, at three months interval for one year and then every six months. Radiological evaluation of graft incorporation was done according to the criteria of Irwin *et al.* (2001) [Table 1].

**Table 1:** Radiological stages of graft incorporation^14^

Stage	Radiolucent zone between the bone cavity and the graft	Intrinsic graft indistinctiveness	Graft margins
I	Present	Distinct	Obvious
II	Indistinct	Indistinct	Hazy
III	Indistinct/disappearance	Indistinct/disappearance.	Obvious incorporation.

Inter-departmental collaboration with the department of Radiology and an opinion from a minimum of two radiologists were obtained to reduce the interobserver bias.

Association of complications and results were analyzed by Fischer's exact test.

## RESULTS

All the 24 patients had an uneventful postoperative recovery. The average follow-up was 18 months (12-36 months). In all except three cases, (*P*-value = 0.003) operative wound healing proceeded uneventfully. Three cases developed superficial wound infection that healed with parenteral antibiotics. In subsequent follow-up, gradual weight-bearing was encouraged. The mean time of full weight-bearing was 14 weeks (range 8-18 weeks). Improvement in the range of movements of the nearby joint was taken as a favorable criterion. All the patients attained a range of movements comparable to or better than their preoperative ranges. The restriction in the range of movements (ROM) preoperatively was (i) Hip: Flexion - 15-90° (mean 30°), Extension - 10-15° (mean 10.7°) (ii) Knee: Flexion - 10-20° (mean 15°), Extension - 10° (mean 10°) and (iii) Shoulder: Abduction - 10-30° (mean-20°), External Rotation - 20-30° (mean 26.3°). The postoperative improvement in the ROM were: (i) Hip: Flexion- mean 31.4°, Extension - mean 10° (ii) Knee: Flexion - mean 15°, Extension - mean 10° and (iii) Shoulder: Abduction - mean 16.3°, External Rotation - mean 20°.

All the cases obtained Stage I incorporation by a mean of 8.7 weeks (range 6-10 weeks). Of them Stage III incorporation was achieved in five cases in mean nine months (range, 6-18 months). While seven cases attained Stage II incorporation in mean of 15.64 weeks (range, 12-18 weeks), nine cases had combined Stages II and III incorporation in different areas, depending on the packing of the cavities. Neither resorption of HA/β-TCP, nor recurrence of the original lesion inside the packed cavity was observed. Extravasation of the implants (HA) was seen in two cases. The combined Irwin *et al.* radiological incorporation and Natarajan *et al.*,^4^ clinical evaluation was used while assessing the results. The results were excellent in nine patients (15.38%), good in 11(69.23%) and fair in four cases (15.38%). Overall 20 cases had a satisfactory outcome (*P* = 0.004), which is significant.

## DISCUSSION

Bone defects caused by resection and curettage of benign bone tumors have been traditionally filled with autogenous cancellous bone graft. Although cancellous graft doesn't provide immediate structural support, it incorporates quickly and ultimately achieves strength equivalent to that of a cortical graft after six to 12 months.^2^ The disadvantages of autogenous bone grafting can be eliminated by biocompatible bone graft substitutes like porous ceramics including HA and β-TCP implants.^6^–^10^ Calcium phosphate ceramics are a group of osteo-conductive materials that are being increasingly used as an alternative to autogenous cortico-cancellous bone graft to fill tumor defects, tibial plateau fracture, spinal fusion, scoliosis surgery, etc.^11^,^12^

The incorporation of HA and β-TCP is determined by two important features of the implant itself: (i) pore diameter, (ii) the porosity or interconnectivity. These porous biomaterials have been shown to have poor mechanical properties but in-growth and overlay of new bone on the trabeculae of these render a more dense structure and once incorporated these become actually stronger than the bone they replaced.^13^ The HA in ceramic and crystalline forms are slow in resorption and bone formation whereas the non-ceramic, non-crystalline form is fast in resorption and bone formation. Beta tri-calcium phosphate is similar to HA but its absorption is faster because of its small grain size and low crystallinity. Beta tri-calcium phosphate is more porous and is resorbed faster than HA making it mechanically weaker in compression. Beta tri-calcium phosphate is an unpredictable bio-degradation product, it has not been popular as a bone graft substitute

Prerequisites for incorporation are: (i) shielding of the biomaterials from excessive loading during bone in-growth by stabilizing the surrounding bone and (ii) apposition of these implants with adjacent viable bone.^14^

Uchida *et al.*, studied clinical results using porous calcium hydroxyapatite blocks and granules to fill bone defects in 60 cases of benign bone tumors after resection.^10^ The implants were well incorporated into the host bone, progressive remodeling of the deformities occurred and no radiologically obvious biodegradation was observed up to five years of follow-up.

Irwin *et al.* retrospectively analyzed 71 consecutive patients who underwent curettage for benign bone lesions and the defects were filled with calcium hydroxyapatite.^15^

We have used calcium hydroxyapatite in 20 cases in the form of blocks and granules and β-TCP granules in four cases. In four cases fibular strut graft was added to pack the cavity along with HA to provide early mechanical stability and in four more cases autogenous iliac cancellous bone graft was added to fill bigger voids and to provide the strength. Autogenous bone graft was added in weight-bearing metaphyseo-diaphyseal areas of the proximal femur and upper end of tibia. We had early radiological incorporation compared to Irwin *et al.*,^15^ as one-third of our cases had either cancellous bone graft or fibular graft supplementation. We had no poor results. The results depended upon stable interface between bone and the HA/β-TCP without micro-motion, proper packing of the defects and the size of the defects. Larger defects took longer time to get incorporated.

Irwin *et al.*,^15^ reported three major and nine minor complications. We had extravasation of HA granules in two cases who are asymptomatic at the latest follow-up and the extravasated HA granules have not under gone biodegradation. Superficial wound infections occurred in three cases, which healed on oral cephalosporins.

Clinical recovery was observed before the radiological recovery with both HA and β-TCP in our series similar to others' series.^16^ After three to five months (mean four months), HA implants showed an increased density with indistinct margins. The β-TCP on the other hand lost the granular radiographic appearance in advance of trabeculae formation. The β-TCP was biologically degraded as healing progressed within the cavitary lesions. The changes began at the edge of the lesion and progressed centrally. On the other hand, HA never completely disappeared and remained un-remodelled even after a long period of implantation.

In our series, histopathological evidence of implant incorporation could not be obtained as none of our patients consented for biopsy. Histological examination in other series showed in-growth of newly formed bone into almost all pore structures of the HA implants.^17^,^18^ There was no conclusive evidence of implant bio-degradation even though presence of histiocytes, multinucleated giant cells and osteoclasts have been reported at the biopsy site. On the other hand, Levin *et al.*,^19^ reported complete resorption of β-TCP in animal study.

Calcium hydroxyapatite and β-TCP alone or mixed with autogenous bone graft are very good substitutes for situations when more graft is required. This is especially true in children as the availability of autogenous bone graft is inadequate in most of the situations while the voids following curettage are large. These implants are bio-compatible and osteo-conductive and conduct new bone formation not only around the implants but also into the pores in a very short period.^20^ However the limitation of our study was that this was a prospective study with short-term results in 24 patients. However, till now no recurrences have been observed.

## References

[1] Giannoudis PV, Dinopoulos H, Tsiridis E (2005). Bone substitutes: An update. Injury.

[2] Finkemeier CG (2002). Bone grafting and bone-graft substitutes. J Bone Joint Surg Am.

[3] Jarcho M (1981). Calcium phosphate ceramics as hard tissue prosthetics. Clin Orthop Relat Res.

[4] Natarajan M, Dhanapal R, Kumaravel S, Selvaraj R, Uvaraj NR (2003). The use of bovine calcium hydroxyapatite in filling defects following curettage of benign bone tumours. Indian J Orthop.

[5] Matsumine A, Myoui A, Kusuzaki K, Araki N, Seto M, Yoshikawa H (2004). Calcium hydroxyapatite implants in bone tumour surgery: A long term follow up study. J Bone Joint Surg Br.

[6] Reddy R, Swamy MK (2005). The use of hydroxyapatite as a bone graft substitute in orthopaedic conditions. Indian J Orthop.

[7] Hulbert SF, Cooke FW, Klawitter JJ, Leonard RB, Sauer BW (1973). Attachment of prostheses to the musculoskeletal system by tissue in growth and mechanical inter locking. J Biomed Mater.

[8] Niwa S, Hori M, Oonishi H, Aoki H, Sawai K (1989). Clinical application of synthetic hydroxyapatite for filling bone defects.

[9] Shinjo K, Makiyama T, Sugiura I, Kondo K, Oonishi H, Aoki H (1989). Clinical application of the hydroxyapatite implants.

[10] Uchida A, Nade S, McCartney E, Ching W (1984). The use of ceramics for bone replacement. J Bone Joint Surg Br.

[11] Bucholz RW, Carlton A, Holmes R (1989). Interporous hydroxyapatite as a bone graft substitute in tibial plateau fractures. Clin Orthop Relat Res.

[12] Holmes RE, Bucholz R, Mooney V (1986). Porous Hydroxyapatite as a bonegraft substitute in metaphyseal defects: A histometric study. J Bone Joint Surg Am.

[13] Shors EC (1999). Coralline bone graft substitutes. Orthop Clin North Am.

[14] Bucholz RW, Carlton A, Holmes R (1987). Hydroxyapatite and tricalcium phosphate bone substitutes. Orthop Clin North Am.

[15] Irwin RB (2001). Coralline-hydroxyapatite as bone substitute in orthopaedic oncology. Am J Orthop.

[16] Shimazaki K, Mooney V (1985). Comparative study of porous hydroxyapatite and tricalcium phosphate as bone substitute. J Orthop Res.

[17] Uchida A, Araki N, Shinto Y, Yoshikawa E, Ono K (1990). The use of calcium hydroxyapatite ceramic in bone tumour surgery. J Bone Joint Surg Br.

[18] Yamamoto T, Onga T, Marui T, Mizuno K (2000). Use of hydroxyapatite to fill cavities after excision of benign bone tumours. J Bone Joint Surg Br.

[19] Levin MP, Getter L, Cutright DE, Bhaskar SN (1975). A comparison of iliac marrow and biodegradable ceramic in periodontal defects. J Biomed Mater Res.

[20] Ogose A, Hotta T, Kawashima H, Kondo N, Gu W, Kamura T (2005). Comparision of hydroxyapatite and beta tricalcium phosphate as bone substitutes after excision of bone tumours. J Biomed Mater Res B Appl Biomater.

